# Developing a Deep Learning Approach for Automated Body Composition Prediction in Newborns Using Ultrasound Images

**DOI:** 10.1109/access.2025.3639889

**Published:** 2025-12-03

**Authors:** KESHI HE, YI LI, HAYOUNG CHO, JULIA HOHENBERG, EMILY NAGEL, SARA RAMEL, KATHERINE A. BELL, JINHEE PARK, DONGLAI WEI, BRYAN J. RANGER

**Affiliations:** 1 Department of Engineering, Boston College, Chestnut Hill, MA 02446, USA; 2 Department of Computer Science, Boston College, Chestnut Hill, MA 02446, USA; 3 Department of Pediatrics, University of Minnesota Medical School, Minneapolis, MN 55454, USA; 4 Department of Pediatrics, Harvard Medical School, Boston, MA 02115, USA; 5 Department of Pediatrics, Brigham and Women’s Hospital, Boston, MA 02115, USA; 6 Connell School of Nursing, Boston College, Chestnut Hill, MA 02446, USA

**Keywords:** Deep learning, body composition, malnutrition, newborn and child health, ultrasound imaging

## Abstract

**Objective:**

Measurements of human body composition such as fat mass (FM) and fat-free mass (FFM) are critical for studying malnutrition and the effects of nutritional interventions. This study introduces research toward a novel ultrasound scanning protocol combined with a deep learning analysis pipeline for predicting body composition.

**Methods:**

We analyzed a clinical dataset of 65 premature infants, consisting of ultrasound images from three anatomical locations (biceps, abdomen, and quadriceps), and ground truth FM and FFM from air displacement plethysmography (ADP). Our investigation focused on determining: 1) the optimal data processing methods for this application; 2) suitable baseline deep learning models for prediction to guide our learning strategy; and 3) the anatomical locations and image regions most predictive of FM and FFM.

**Results:**

We demonstrate that: 1) pre-processing techniques such as denoising, median filtering, and data augmentation enhance performance; 2) by employing a modified EfficientNet-B1 architecture, we achieve fully automatic body composition predictions from ultrasound images; 3) images obtained from combinations of biceps and quadriceps, as well as biceps, quadriceps, and abdomen scanning locations, resulted in mean absolute percent error (MAPE) values of 26.1% and 25.32%, respectively. Finally, sensitivity analysis shows that FM and FFM prediction are influenced by different body parts, as well as adipose and muscle tissue thickness.

**Conclusion:**

This study represents the first demonstration of deep learning for automated human body composition prediction from ultrasound images and lays a critical foundation for a novel ultrasound scanning and interpretation protocol to assess malnutrition.

## I. INTRODUCTION

Human body composition measures, such as fat mass (FM) and fat-free mass (FFM) play a critical role in studying malnutrition and assessing changes due to nutritional, therapeutic or behavioral interventions [1]. Nutritional assessment in infancy is crucial, as this is a critical period for growth and development during which disruptions can lead to lifelong consequences [2]. However, body composition measurement techniques such as dual-energy X-ray absorptiometry (DXA), computed tomography (CT), and air displacement plethysmography (ADP) require specialized staff and equipment, which are costly and not widely available [3].

Ultrasound imaging presents a promising alternative capable of distinguishing between adipose tissue and skeletal muscle [4]. Its accessibility has recently increased due to the rise in availability of low-cost portable ultrasound systems [5]. However, fully automated body composition prediction from ultrasound images has yet to be developed.

Deep learning [6] is a promising tool for automating clinical diagnosis for various disorders [7], [8], [9], [10], [11], [12], [13], [14], [15], [16], [17] and has been widely used for segmentation, classification, and detection in medical image analysis [18], [19], [20]. Currently, most research on deep learning-based human body composition prediction is based on CT data in adult subjects [21], [22], [23], [24]. For instance, Weston et al. [21] proposed a fully automated convolutional neural network (CNN) model based on U-Net for segmenting the abdomen from CT images to quantify body composition, achieving dice scores exceeding 0.9. Koitka et al. [22] developed a 3D semantic segmentation CNN, with an average dice score of 0.9553 and the intra-class correlation coefficients for subclassified tissues above 0.99. Xu et al. [24] developed a fully automatic pipeline to derive body composition measurements from routine lung screening chest low-dose CT. Despite these advances, CT is not an ideal option for routine body composition measurement, particularly in infants, due to its relatively high cost and exposure to ionizing radiation. In contrast, ultrasound presents a promising alternative for this application, given its inherent low-cost, portability and lack of ionization radiation. Recently, Nagel et al. [25] demonstrated encouraging results when studying body composition of premature infants using manual tissue thickness measurements obtained from ultrasound.

**FIGURE 1.**
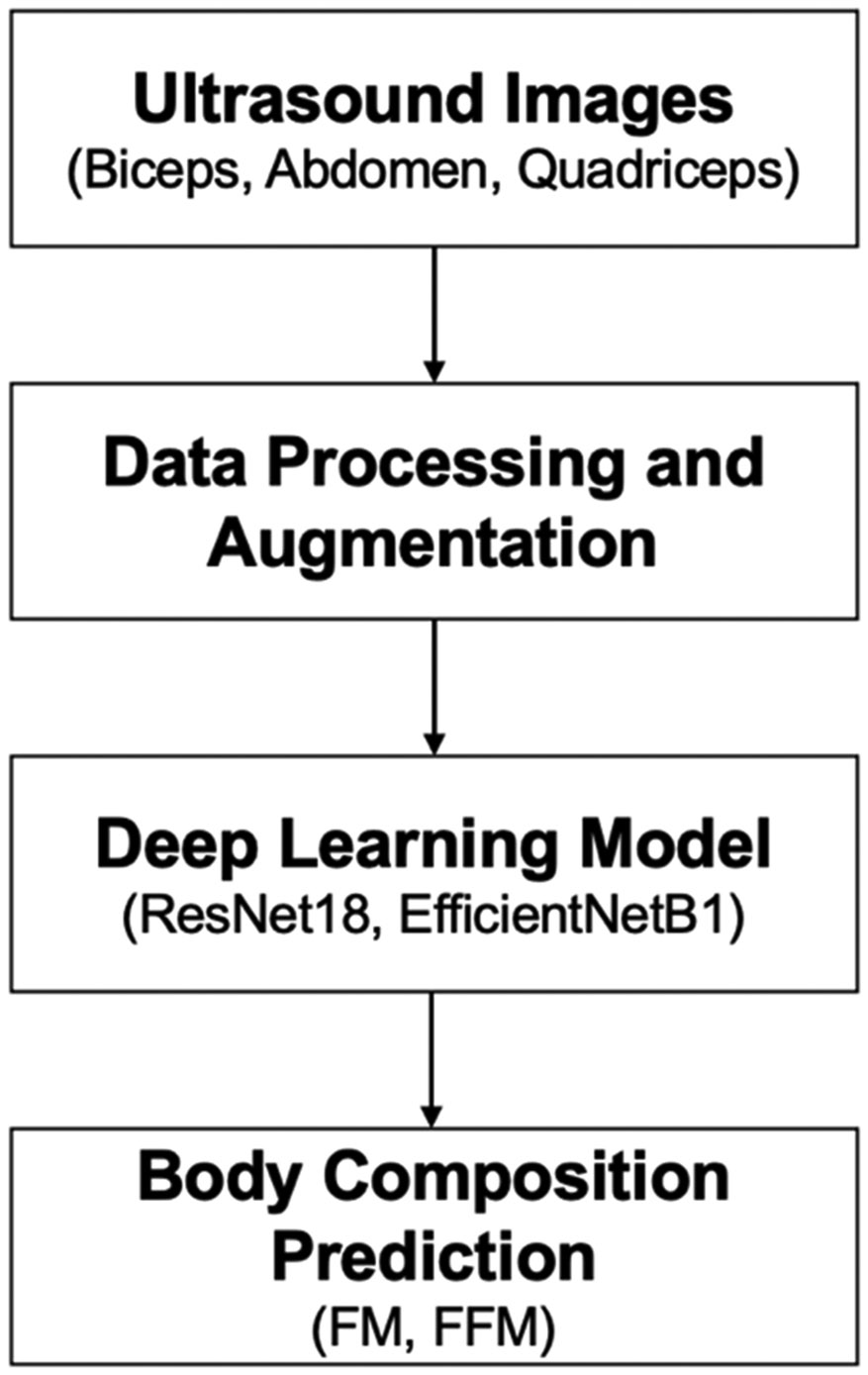
Flowchart of the deep learning approach used to predict body composition from ultrasound images.

Our ultimate vision is to apply deep learning interpretation techniques to ultrasound images and develop predictive algorithms for important nutritional and growth metrics (Fig. 1). Toward this goal, we present research related to the development of a novel ultrasound scanning protocol and analysis pipeline, which will ultimately be deployed for computer-assisted intervention (CAI), in which nutritional interventions may be informed by computer-based tools and methods. Our work is driven by the following research questions: (1) Data pre-processing: what data processing is most suitable for this application? (2) Data analysis: which baseline deep learning models provide predictions to inform our learning strategy? (3) Data acquisition: which anatomical locations and image regions are most predictive of FM and FFM? Following this, we conducted sensitivity analysis to quantitatively evaluate how the prediction performance of FM and FFM can be influenced by different human body parts as well as muscle and adipose thickness. To address these questions, we analyzed data collected as part of a previous study on premature infants conducted at the University of Minnesota Medical Center. The dataset comprises of ultrasound images collected from three anatomical locations (leg, arm, and abdomen), ground truth FM and FFM from air displacement plethysmography (ADP), and clinical data extracted from patient records.

The contributions of this paper are summarized as follows: (1) We identified the most suitable data preprocessing techniques, including median filtering, denoising, and image augmentation, which can enhance prediction performance; (2) We demonstrate, for the first time, end-to-end automatic estimation of human body composition, finding that EfficientNet-B1, when fine-tuned and trained with the mean absolute error (MAE) loss function, provided the best baseline among the models evaluated; (3) We found that combinations of biceps and quadriceps images, as well as combinations of biceps, abdomen, and quadriceps, yielded the lowest mean absolute percentage error (MAPE) in predictions. Finally, sensitivity analysis revealed that different body parts affect the prediction performance of FM and FFM which is also influenced by different muscle and adipose thicknesses. To the author’s knowledge, this study represents the first demonstration of utilizing deep learning techniques for automated human body composition prediction from ultrasound images. Therefore, our results provide important guidelines for optimizing a novel ultrasound scanning and analysis pipeline to assess malnutrition.

## II. MATERIALS AND METHODS

### A. EXPERIMENTAL DATA DESCRIPTION

All data were collected as part of a previous study [25] conducted at the University of Minnesota Medical Center. A total of 65 premature infants were recruited following an IRB-approved protocol, with informed consent obtained from their parents. The infants were born between 25 and 34 (+6) weeks’ gestational age, were medically stable at time of measurement, and had not undergone major surgeries or received significant medical diagnoses. Following clinical data collection, all data were de-identified, thus eliminating the need for additional IRB approval for analysis.

The dataset comprises ultrasound images, gold standard body composition, and clinical measures. Ultrasound images of infant biceps (brachii and brachialis), abdomen (rectus abdominis), and quadriceps (rectus femoris and vastus intermedius) were collected using a B-mode ultrasound imaging system (NextGen LOGIQ e R7, GE Medical Systems, Chicago, IL). These anatomical locations were chosen since they are representative of muscle and adipose tissue distribution at different regions of the body and are easily accessible for scanning. Ultrasound measurements of adipose and muscle thickness were conducted by a clinician using the system’s built-in measurement tools. Gold standard body composition values, including FM and FFM, were generated via ADP using a PeaPod system (Cosmed, Ltd, Concord, CA). Ultimately, we curated a representative dataset including 414 ultrasound images of arm, leg, and abdomen with triplicate measurements from 46 infants. This secondary analysis excluded 19 subjects that lacked complete ultrasound image or clinical data. The clinical data included measurements such as body composition, body weight, height, age, and gender, as detailed in Table 1.

### B. DATA PRE-PROCESSING

**FIGURE 2.**
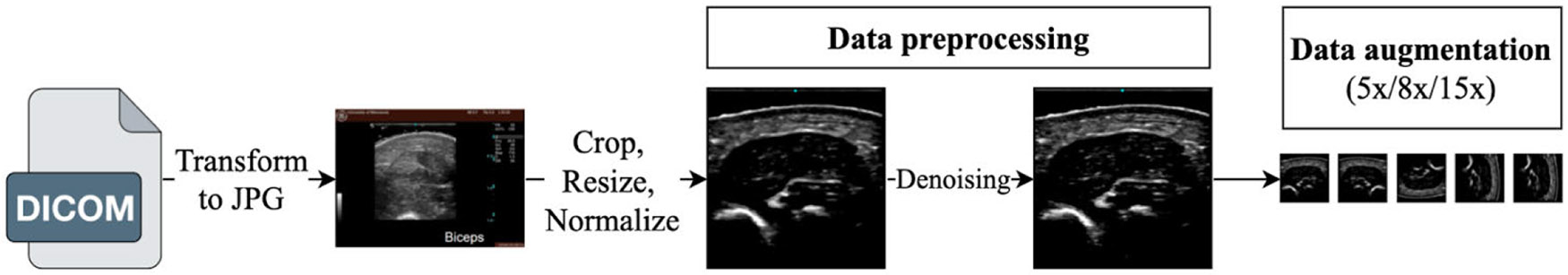
Flowchart showing pre-processing steps of ultrasound images.

The ultrasound image data underwent a series of preprocessing steps to prepare it for analysis. First, the data was converted from DICOM to.jpg format. The original images were then cropped to remove the background. Subsequently, a series of image preprocessing approaches were applied as follows: (1) raw cropped ultrasound images were resized to 256 × 256; (2) resized ultrasound images were denoised using a median filter of size 5 to enhance ultrasound image quality; (3) ultrasound images were normalized with a mean and standard deviation of 0.5; and (4) the dataset was augmented from 414 images (9 images for each of the 46 infants) by a factor of five using vertical flipping, horizontal flipping, and random rotation to improve the scale and diversity of the ultrasound image dataset in order to mitigate the risk of model overfitting. Fig. 2 depicts all steps associated with the overall ultrasound images preprocessing.

### C. DATA ANALYSIS: MODEL DESIGN CHOICES FOR DEEP LEARNING PIPELINES

Preprocessed ultrasound images, along with FM and FFM derived from gold standard ADP [2], were utilized for model training and evaluation. The trained model was designed to automatically generate end-to-end predictions of FM and FFM. The experimentation involved two models, two optimizers, four loss functions, four data processing, and two methods, as summarized in Table 2.

For the initial analysis, ResNet18 and EfficientNet-B1 models were selected due to their well-established balance of computational efficiency, prediction accuracy, and training stability, which are key considerations for regression tasks such as a body composition prediction. While other architectures like U-Net excel in image segmentation, our study focuses on direct regression from images (i.e., B-mode ultrasound image-to-FM/FFM value regression), making EfficientNet-B1 and ResNet18 suitable for our task. To evaluate model performance across varying data conditions, we constructed 21 subsets from our data sets using 7 different combinations of data, and 3 different numbers of images from each muscle. For instance, one subset consisted of both biceps and quadriceps with 2 images withdrawn from each muscle.

**FIGURE 3.**
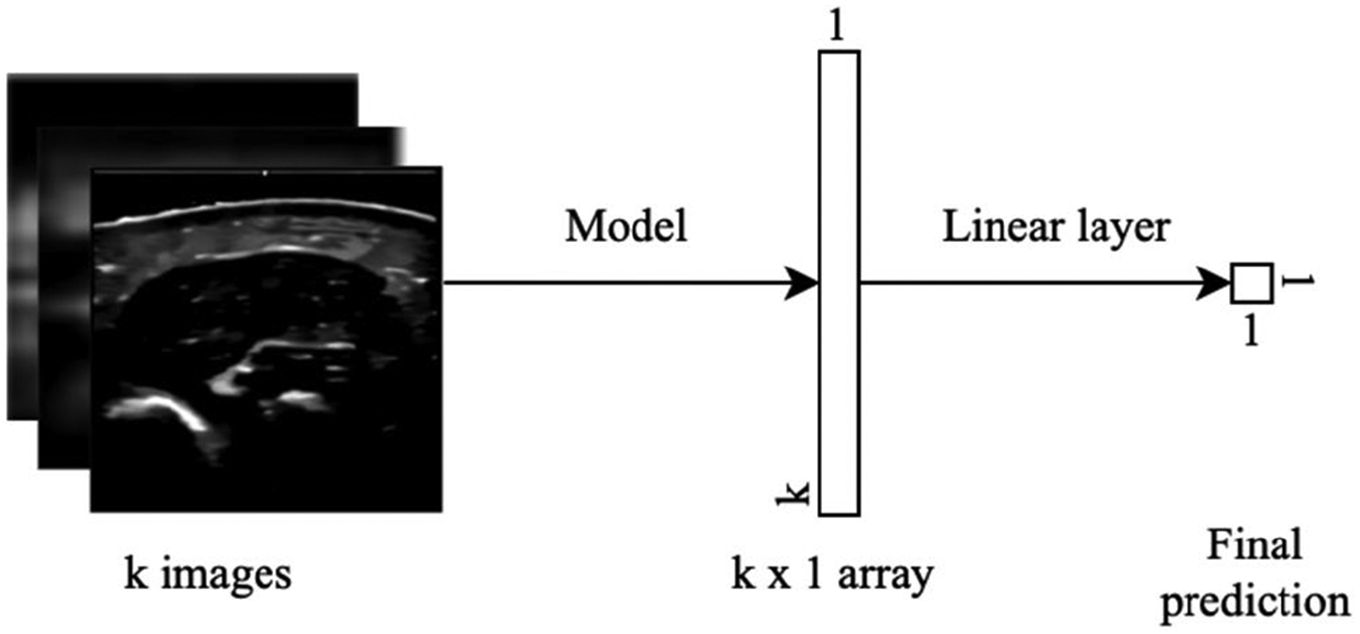
Deep learning model design for human body composition prediction.

For the ResNet18 and EfficientNet-B1 regression models, each ultrasound image was processed to extract model features, which were then aggregated through averaging. These aggregated features were subsequently passed to a linear layer for regression. The overall deep learning model design is shown in Fig. 3.

It is shown that the first linear layer was applied on an individual image basis, concatenating the 256 × 256 matrix of features to a single float. After obtaining a value for each *n* image, we performed a late fusion technique in which we concatenated *n* numbers in each batch into a single float, which predicted either FM or FFM.

#### 1) MODEL TRAINING

Our dataset was split into training, validation, and testing sets in a 70-20-10 ratio, resulting in 32 infants for training, 9 for validation, and 5 for testing. Each patient had 3 abdomen images, 3 biceps images, and 3 quadriceps images, along with 36 augmented images, leading to a batch size of 45 images. Various combinations and numbers of images from each muscle were tested, and hyperparameters were adjusted to optimize model performance. Training and hyperparameter tuning were iterated to enhance performance. The study focused on human body composition labels, including fat mass (FM) and fat-free mass (FFM). Different loss functions were employed, such as MAPE, mean square error (MSE), mean absolute error (MAE), and custom loss function composed of 60% MSE and 40% MAE. Both the Adam optimizer algorithm [28] and the Stochastic Gradient Descent (SGD) [29] were used for model training. A mini-batch size of 1 and a fixed learning rate schedule of 0.001 were employed, with 40 epochs identified to be the most effective. Minimal performance improvement was observed beyond 40 epochs of training. To prevent the overfitting of the deep learning model, the following techniques were employed: 1) multiple dropout layers with a rate of 0.1; 2) leaky ReLU with a 0.1 negative slope applied twice in each convolution block; and 3) L2 regularization.

#### 2) MODEL EVALUATION

Common evaluation metrics were used to quantify the performance of model regression tasks. In this study, the predictive model was evaluated using the MAPE, given its intuitive interpretation in terms of relative error.

#### 3) MODEL IMPLEMENTATION

All deep learning models were programmed and implemented by using Python 3.9 and the PyTorch deep learning library. The computational tasks were performed on a Dell G15 Gaming Laptop equipped with an NVIDIA^®^ GeForce RTX^™^ 3060 GPU and an 11th Generation Intel^®^ Core^™^ i7-11800H processor. Additionally, a T4 GPU hosted on Colab was utilized to accelerate the training processing. The model computation typically lasted 0.8 to 1.0 hours, varying based on the computational resources and the image preprocessing techniques. During the computation, both model training and testing loss curves experienced the rapid initial decreases, followed by gradual and eventual stability with minimal variation after 40 epochs.

In order to verify the necessity of deep learning models, we employed traditional feature-based regression methods. Specifically, we utilized Histogram of Oriented Gradients (HOG) [30] and Scale-Invariant Feature Transform (SIFT) [31] with Bag of Visual Words (BoVW) [32] for feature extraction. These techniques were combined with Random Forest regression to predict body composition from ultrasound images. The performance of these models was then compared to the results obtained using deep learning models (i.e., EfficientNet-B1) to assess the superiority of modern deep learning approaches over traditional methods.

### D. DATA ACQUISITION: DETERMINING WHICH ANATOMICAL LOCATIONS OPTIMIZE PREDICTION

**FIGURE 4.**
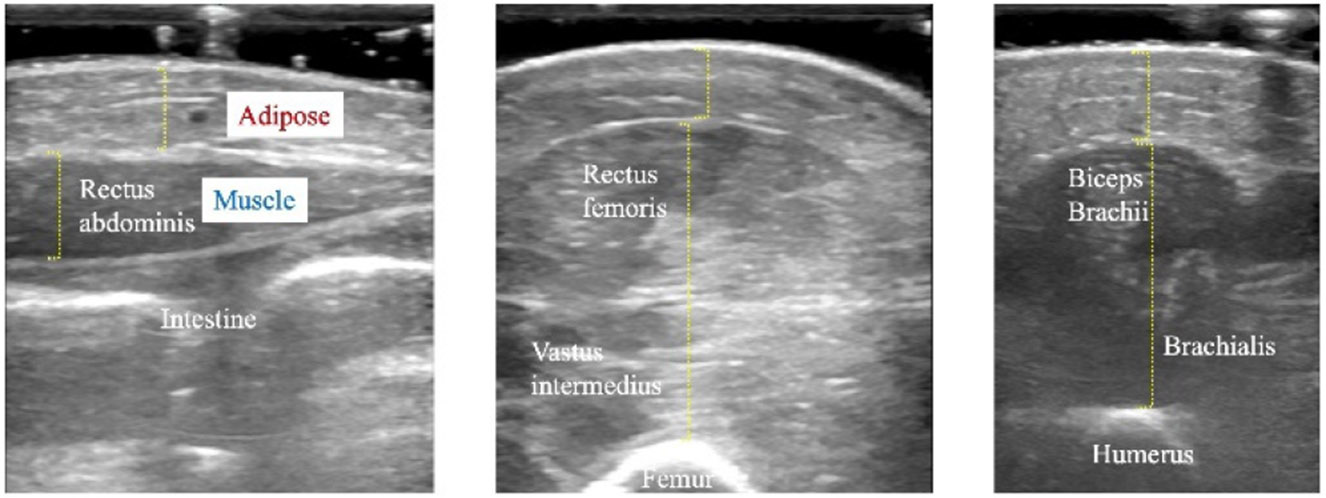
Clinical ultrasound image measurement of abdomen, biceps, and quadriceps of the premature infant. *Left*: abdomen, *Middle*: biceps, and *Right*: quadriceps.

Currently, there is no standardized experimental protocol for collecting ultrasound images or videos to assess human body composition in the literature. We collected relevant experimental data, with ultrasound images were obtained from three anatomical locations on a newborn: leg (quadriceps), arm (biceps), and abdomen. The clinically measured ultrasound images of abdomen, biceps and quadriceps of the premature infant are shown in Fig. 4. When considering clinical deployment, it is imperative to determine which anatomical location and image regions, or combinations thereof, provide an optimal prediction of body composition measures. This will ultimately lead to a streamlined protocol for collecting pertinent data for this clinical application.

In this study, we systematically evaluated the prediction performance with the following combinations of ultrasound images: biceps only (B), quadriceps only (Q), abdomen only (A), biceps + quadriceps (BQ), abdomen + biceps (AB), abdomen + quadriceps (AQ), and biceps + quadriceps + abdomen (BQA).

### E. QUALITATIVE ANALYSIS OF MODEL ERRORS

To visualize the decision-making process of the EfficientNet-B1 model with fine-tuning, we applied Gradient-weighted Class Activation Mapping (Grad-CAM) [33] to generate heatmaps that highlight spatial regions within the input images that contribute most significantly to the model’s predictions. The Grad-CAM computes the gradients of the output with respect to the feature maps of a specified convolutional layer, producing a localization map that reflects the relative importance of different regions. In this study, Grad-CAM heatmaps were generated for all three anatomical regions using activations from the final convolutional layer of the EfficientNet-B1 architecture.

### F. STATISTICAL ANALYSIS

Gaussian process (GP) [34] was applied to predict PerFM and PerFFM for all six body parts. The data was split into 80% train, 10% test, and 10% validation. Since there were no clear outliers in the 12 plots of BM vs PerFM, BM vs PerFFM, BA vs PerFM, etc, root-mean-square error (RMSE) and mean squared error (MSE) were used rather than (mean absolute error) MAE.

## III. RESULTS

### A. DATA PRE-PROCESSING

To determine a suitable data preprocessing pipeline for the deep learning model, a series of ultrasound data preprocessing steps were conducted as follows:

#### 1) DENOISING

Among various denoising methods, such as stochastic denoising, we found that median filter was most effective for our dataset. We experimented with different pixel sizes for the median filter, and determined that a pixel size of 5 produced the best results. Larger or smaller sizes either did not improve performance significantly or resulted in performance degradation.

#### 2) AUGMENTATION

We compared various approaches to image augmentation. Augmenting each image within the dataset (without adding new ground truth data) proved more effective than augmenting the entire dataset by adding new sets of augmented data with corresponding ground truth. Specifically, augmenting each image five times provides the best balance between computational efficiency and performance improvement.

#### 3) RESIZING

We tested multiple resizing configurations, including 512 × 512, 256 × 256, 128 × 128, and 64 × 64, to identify the most suitable balance between computational efficiency and model performance. Our experiments indicated that 256 × 256 provided the optimal trade-off. This resolution allowed us to maintain high prediction performance while reducing computational demands compared to higher resolutions. Although 512 × 512 resulted in marginal improvements in performance, it introduced a substantial computational burden, making it less practical for efficient model training. On the other hand, 128 × 128 and 64 × 64 led to a noticeable decline in performance. Consequently, we found 256 × 256 to be the most effective choice, offering a good balance between computational efficiency and predictive accuracy, and is consistent with common practices in ultrasound image analysis.

#### 4) NORMALIZATION

We experimented with different normalization techniques such as min-max normalization and mean normalization. The standard normalization to a mean of 0.5 and a standard deviation of 0.5 proved to be the most effective approach.

Our final pre-processing strategy consists of median filtering (pixel size 5), resizing images to 256 × 256 pixels, and standard normalization. This combination provides significant performance improvements over raw data. Additionally, we found that median filtering alone improved prediction accuracy, and applying image augmentation (augmenting each image five times) alone also improved performance. However, combining both median filtering and augmentation did not lead to further significant improvements, indicating that each technique independently contributes to performance enhancement.

### B. DATA ANALYSIS: MODEL DESIGN CHOICES FOR DEEP LEARNING PIPELINES

For FM prediction, the traditional model based on HOG features achieved an MAE of 0.1061 kg, MSE of 0.0155 kg^2^, RMSE of 0.1247 kg, and MAPE of 116.23%. Similarly, the SIFT-based model utilizing BoVW has comparable results, with an MAE of 0.1101 kg, MSE of 0.0171 kg^2^, RMSE of 0.1309 kg, and MAPE of 125.21%. Therefore, both models displayed limited ability to capture variability across infants, with predictions consistently hovering around a constant FM of approximately 0.2 kg.

Table 3 presents the FM prediction results of various models using various optimizers and loss functions. In contrast, the deep learning models outperformed these traditional approaches across all error metrics. EfficientNet-B1 with fine-tuning and MAE loss function exhibited the best performance, achieving an MAE of 0.0455 kg and a MAPE of 25.32%. Therefore, EfficientNet-B1 with the MAE loss function was selected for further performance evaluation across different muscle combinations.

### C. DATA ACQUISITION: ANATOMICAL LOCATIONS THAT ARE MOST PREDICTIVE OF FM AND FFM

Utilizing EfficientNet-B1, we compared all combinations of acquisition protocols. Results are summarized in Table 4. As shown, BQ and BAQ resulted in the lowest MAPE values when predicting body composition, which indicates that they are most predictive of FM and FFM.

### D. ABLATION STUDIES

We conducted 50 independent runs of each model configuration on the same test dataset, recording the MAPE for each run. Subsequently, we performed one-sided paired t-tests to compare the distributions of MAPE values across model configurations. We calculated p-values to evaluate whether one configuration consistently yielded lower MAPE values compared to another. Table 5 presents these p-values, where a value close to 0 indicates that the MAPE values in the row are significantly lower than those in the columns, while a value close to 1 suggests the opposite. Based on this analysis, the best configuration is EfficientNet-B1 with fine-tuning and the mean absolute error (MAE) loss function, followed by ResNet18 with linear probing and the mean squared error (MSE) as the second-best configuration. The top two configurations are not significantly different from each other but are significantly different from the others.

In addition, we also analyzed the effect of the training strategy and loss function on model performance. For ResNet18, Linear Probing showed statistically significant improvements over Fine-Tuning, which suggests that linear probing may better capture the relevant features for this particular architecture. In contrast, for EfficientNet-B1, fine-tuning outperformed Linear Probing, demonstrating the importance of adjusting the weights in this model to achieve optimal performance. Interestingly, the choice of loss function, whether MAE or MSE, did not show consistent performance differences, indicating that both loss functions are equally effective in optimizing the models for this task.

The results further highlight the superiority of the EfficientNet-B1 model with fine-tuning and MAE loss function, as evidenced by significantly lower MAPE values. This configuration consistently outperformed all other models, suggesting that it is the most effective choice for predicting human body composition from ultrasound images. The findings also reveal that, fine-tuning is a more effective strategy for certain models, while Linear Probing is a more appropriate strategy for others, which indicates that the effectiveness of training strategies is architecture-dependent.

**FIGURE 5.**
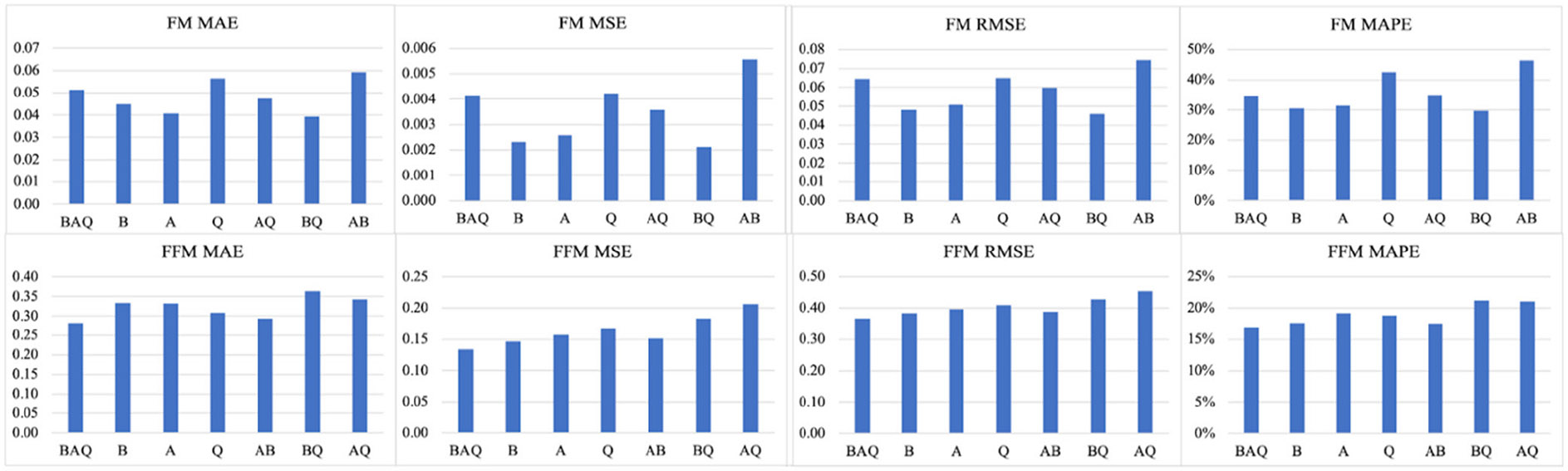
Comparison of human body composition measurements (FM and FFM) across various combinations of body parts.

Fig. 5 illustrates the systematic comparisons of all four evaluation metrics for both FM and FFM across seven combinations of human body parts. For FM prediction, the model trained with the combination of biceps and quadriceps outperforms those trained with other datasets. For FFM prediction, the model trained using all three body parts (biceps, abdomen, and quadriceps) achieves the best performance.

### E. GRAD-CAM VISUALIZATION

**FIGURE 6.**
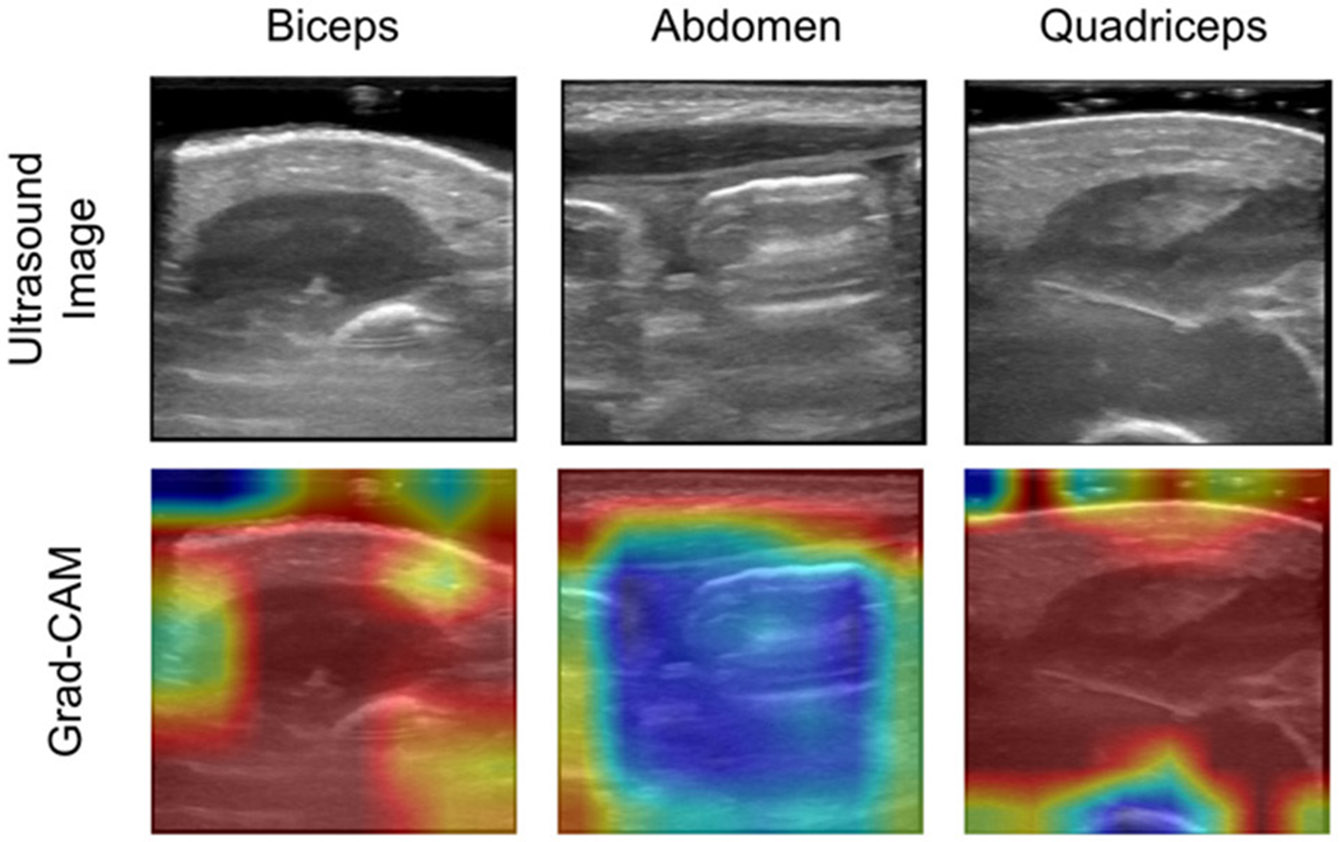
The top row of images shows representative ultrasound images of the biceps, abdomen, and quadriceps. The bottom row shows Grad-CAM results of the contributory regions in the ultrasound images of abdomen, biceps and quadriceps. Warmer colors (reds) indicate higher importance in the model’s prediction, while cooler colors (blue) indicate lower importance in the model’s prediction.

Fig. 6 displays Grad-CAM visualizations for a representative subject, highlighting the regions within the ultrasound images of the abdomen, biceps, and quadriceps that most influenced the model’s predictions. Regions with warmer colors (e.g., red) denote higher relevance, while cooler colors (e.g., blue) indicate lower relevance in the prediction process. The feature maps reveal a consistent emphasis on muscular regions, with muscle tissue generally receiving greater attention than subcutaneous fat or underlying bone structures such as the femur and humerus. In particular, the abdominal images show that subcutaneous tissue is contributing more to the model output as compared to deeper tissues.

### F. SENSITIVITY STUDIES

**FIGURE 7.**
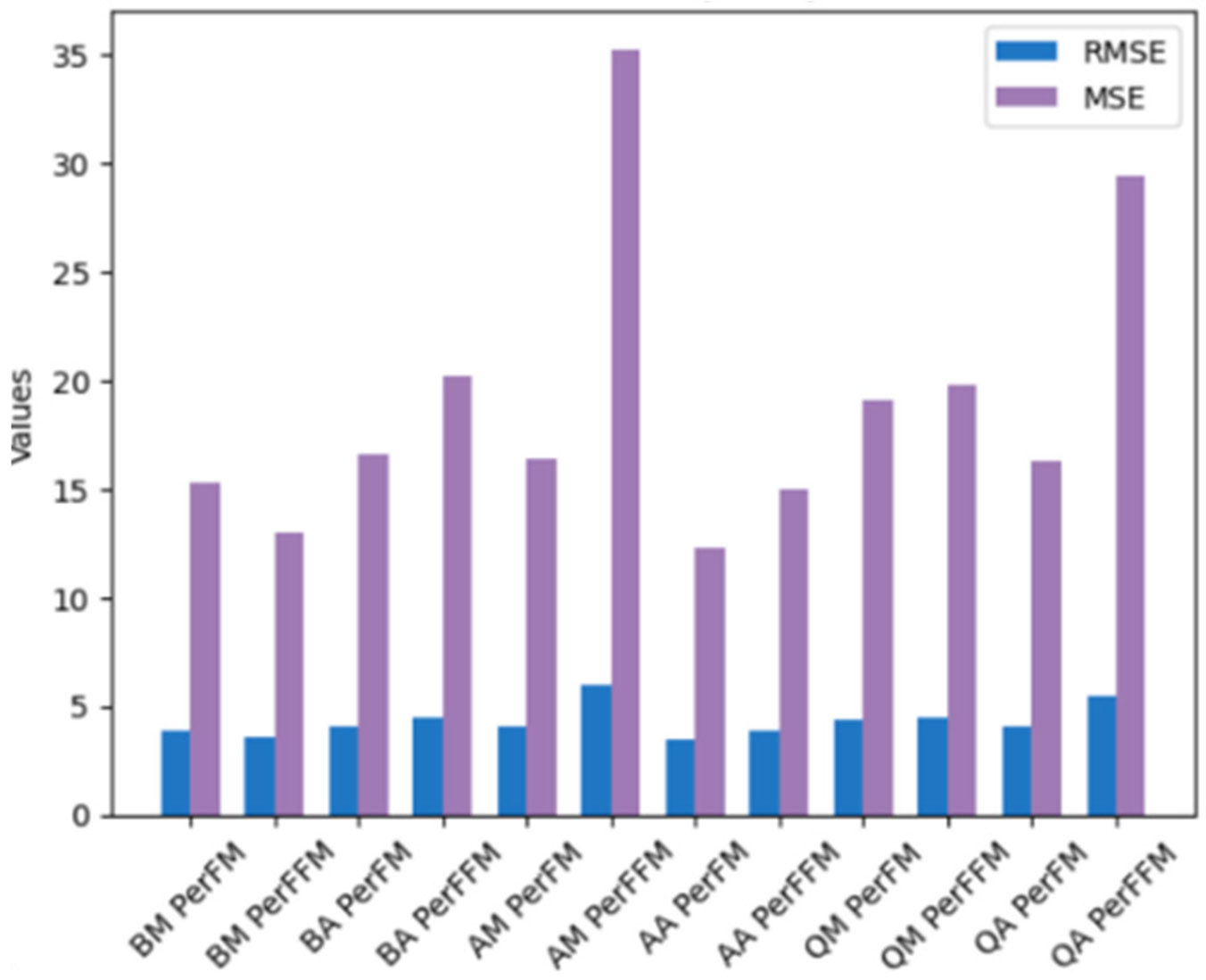
The histogram showing performance of utilizing adipose tissue/muscle thickness to predict PerFM/PerFFM (average measurement abbreviation – BM: biceps muscle, BA: biceps adipose, AM: abdominal muscle, AA: abdominal adipose; QM: quadriceps muscle, and QA: quadriceps adipose).

We conducted a sensitivity analysis to evaluate the human body composition prediction performance based on muscle and adipose tissue thickness measurements. The performance of PerFM and PerFFM prediction for each body part is shown in Fig. 7.

RMSE and MSE were employed instead of MAE due to the absence of clear outliers in the resulting plots. It can be observed that prediction performances for FM and FFM vary across different body parts. RMSE ranges from 3.51 to 5.94, while MSE ranges from 12.29 to 35.24 across all cases. Notably, BM PerFM/PerFFM and AA PerFM/PerFFM prediction exhibit the best performance. Additionally, our findings reveal that, adipose tissue thickness-based PerFM prediction has a lower RMSE than muscle thickness-based PerFM prediction for abdomen and quadriceps images, whereas muscle thickness-based PerFFM prediction has a lower RMSE than adipose tissue thickness-based PerFFM prediction for biceps and quadriceps images.

## IV. DISCUSSION

Ultrasound has significant potential applications for nutritional evaluation and analysis. For instance, it can be used for assessing the nutritional status of older adults [35], conduct nutritional assessment of children with nephrotic syndrome [35], and perform morpho-functional assessment of disease related malnutrition [37]. Unlike methods such as CT scans, ultrasound is radiation-free and more cost-effective. However, ultrasound has yet to be utilized for the automated estimation of human body composition, which is a critical metric for nutritional assessment. To our knowledge, this research is the first to apply deep learning-based image analysis for this purpose.

Ultrasound imaging is a promising tool for predicting body composition. However, there are no standard experimental protocols for conducting such measurements in the literature. In addition, no publicly available datasets exist for this application, which precludes external validation at this stage of the work. Our ultimate goal is to develop an automated ultrasound tool for body composition prediction that could ultimately be deployed for point-of-care nutritional assessment. In this paper, we pioneer the use of ultrasound imaging to develop a deep learning-based pipeline for automated human body composition prediction.

First, we conducted an analysis of ultrasound data using deep learning-based techniques to determine effective preprocessing methods, including median filtering, denoising, and image augmentation. These steps enhanced both the computational efficiency and the prediction performance of our deep learning models. This data preprocessing pipeline may serve as a foundation for further developing deep learning models for this and other musculoskeletal ultrasound applications.

Then, the comparison between traditional feature-based models and deep learning approaches highlights the clear advantages of the deep learning approaches in predicting FM. Both HOG and SIFT with BoVW models resulted in high error metrics, with MAPE values exceeding 100%, and struggled to capture the variability in FM across different infants, and consistently predicted values close to a constant. In contrast, the deep learning models showed significant improvements in prediction accuracy. Particularly, EfficientNet-B1 with fine-tuning is well-suited as a deep learning baseline model, providing valuable insights for optimizing learning strategies for human body composition prediction from ultrasound. Moreover, we also explored the use of GPR as part of the sensitivity studies to assess its potential for handling ultrasound image data for this task. GPR has shown promise in various ultrasound-based applications, such as human leg localization [38] and gesture classification [39], due to its ability to provide robust predictive performance alongside uncertainty estimation. Future work may further investigate the integration of GPR with deep learning models to leverage its uncertainty estimation capabilities, particularly in clinical settings where model confidence is critically important.

In addition, we conducted a focused comparison of data acquisition protocols, specifically evaluating the contributions of images from different body parts to body composition prediction. By implementing EfficientNet-B1, we show that BQ and BAQ had the lowest MAPE values for prediction of FM compared to the other combinations of images. In contrast, all other combinations, including those for FFM predictions, were considerably higher. Therefore, it can be concluded that anatomical locations BQ and BAQ, along with their respective image regions are most predictive of FM and FFM. These findings are consistent with previous experimental studies in the literature, which indicate that specific body regions were commonly used for infants (0-1 years) to assess the whole-body composition. In those studies, biceps brachii have a strong relationship with body mass index [40] and total body muscle mass [41]. While these results merit further investigation, they suggest that particular combinations of images can lead to improved predictions, potentially streamlining clinical protocols.

Furthermore, the Grad-CAM visualizations provide valuable insight into the model’s decision-making process by highlighting the anatomical regions that contribute most significantly to its predictions. The heatmaps display that muscle tissue such as biceps and quadriceps significantly contributes to the model’s predictions. Conversely, subcutaneous fat and bone structures (e.g., femur and humerus) contribute less to the model’s predictions. Notably, the abdominal region showed a distinct separation between muscle and fat, suggesting that the model places greater emphasis on superficial tissue than deeper tissue for body composition prediction. In conclusion, these results reveal the model’s reliance on key anatomical features and offer a more transparent understanding of how different human body parts in ultrasound images affect body composition predictions.

Our sensitivity analysis also examined the influence of different anatomical regions on prediction performance for FM and FFM. BM PerFM/PerFFM and AA PerFM/PerFFM achieve the best performance. The prediction performance metrics, RMSE and MSE, of PerFFM are higher than that of PerFM for most cases. Our findings indicate that adipose tissue thickness-based predictions for PerFM exhibit a lower RMSE compared to muscle thickness-based predictions for abdomen and quadriceps images. Conversely, muscle thickness-based predictions for PerFFM demonstrate a lower RMSE than those based on adipose tissue thickness for biceps and quadriceps images.

In this study, we successfully establish the feasibility of using deep learning for end-to-end prediction of human body composition from ultrasound images, focusing exclusively on imaging data to develop and evaluate the effective data processing pipeline. Our findings provide a foundational benchmark and a methodological contribution by proposing an effective baseline model and an optimized data processing pipeline for this task. Overall, this study presents a novel deep learning approach for fully automated, ultrasound-based body composition prediction and then validates its feasibility, which lays the foundation for future research and broader clinical applications. Our preliminary results exhibit great potential for an end-to-end deep learning solution for automated ultrasound-based body composition prediction, potentially enhancing point-of-care assessments of malnutrition. While the study focuses on newborns, these techniques have broader applications, including the diagnosis of obesity, diabetes, and age-related musculoskeletal changes, as well as other nutritional evaluations.

However, we note several limitations. We firstly acknowledge that the dataset used in this study is limited to 65 premature infants, with only 46 infants having complete data across all relevant modalities. This relatively small sample size is a key limitation, particularly for training deep learning models, which typically require larger and more diverse datasets to achieve robust generalization. Therefore, we recognize the preliminary nature of these findings, which demonstrate the feasibility of predicting body composition from ultrasound and provide a foundation for future research. Nevertheless, these results offer valuable insights for guiding subsequent studies and highlight the need for larger-scale data collection efforts to validate and refine the proposed approach. These insights are crucial for designing and optimizing experimental protocols, and for establishing a baseline for future research. An additional limitation of this study is the absence of an external validation set, which restricts our ability to assess the generalizability of the model across different populations and clinical settings. The dataset used in this work was collected from a single institution, and access to external datasets was not available at this stage of the research. Considering the primary aim of this study was to establish the feasibility of using deep learning for ultrasound-based prediction of human body composition and to develop an effective data processing pipeline, within this context, our findings serve as a proof-of-concept, and demonstrates the potential of the proposed approach.

On the other hand, when considering the successful deployment of such models in the future, several key challenges must be addressed as follows: 1). Optimizing real-time inference speed will be essential for point-of-care applications, while maintaining prediction accuracy; 2). Enhancing the model’s interpretability with techniques such as Grad-CAM will be crucial for clinician trust and adoption; 3). Obtaining regulatory approval and ensuring compliance with safety standards will be necessary steps for clinical use; 4). Ethical considerations, including the role of automated decision-making in supporting rather than replacing clinician judgment, will also be integral to future development.

In upcoming studies, we will build on these results to develop novel deep learning architectures for the body composition prediction task, incorporate additional image analysis techniques (e.g., segmentation of various anatomical structures, such as fat and muscle layers), and utilize non-image clinical characteristic data (e.g., sex, birth weight, and age) as supplementary model inputs. Then, in order to improve our datasets, we will expand data collection to include multiple sites and more diverse populations, which will be essential for conducting external validation and ensuring broader applicability of the model. We are currently conducting clinical studies at two academic medical centers in East Africa and North America, where we are gathering additional ultrasound data and clinical metrics from diverse populations. We anticipate that aggregating these new datasets with our existing data will enhance our deep learning pipeline’s robustness and generalizability. Although this study centers on preterm infants, we will also expand our research to include full-term infants to evaluate consistency across populations. Furthermore, future studies will investigate outliers in model predictions and extend our analysis to other high-risk infant groups, thereby broadening the impact of our findings. Finally, we will explore the deployment of the deep learning models in practical scenarios, with the potential to enhance clinical decision-making and patient care.

## V. CONCLUSION

In this study, we curated a representative experimental dataset of ultrasound images alongside gold standard body composition values from ADP, and developed a deep learning-based pipeline for predicting human body composition. The key contributions of this paper are as follows: (1) The implementation of median filtering, denoising, and image augmentation achieved a balance between computational efficiency and prediction performance; (2) Among the models evaluated, EfficientNet-B1 with fine tuning and trained using the MAE loss function demonstrated the most promising baseline performance; (3) The combinations of biceps and quadriceps images, as well as biceps, abdomen, and quadriceps, resulted in predictions with the lowest MAPE. Finally, sensitivity analysis indicates that the prediction performance of FM and FFM is influenced by various body parts, as well as muscle and adipose thicknesses. Our findings provide critical guidelines for optimizing a novel ultrasound scanning and interpretation protocol for predicting human body composition, with significant implications for the clinical assessment of infant malnutrition.

## Figures and Tables

**TABLE 1.** Descriptive characteristics of enrolled infants.

Variable	Mean	Standard Deviation
GA_birth	32.04	2.05
PMA_study visit	35.18	1.23
Weight_visit	2.15	0.39
Length_visit	43.90	2.41
PerFM	8.82	4.17
PerFFM	91.18	4.17
FM	0.19	0.10
FFM	1.96	0.35
PMA_discharge	37.51	1.96
PMA_PO start	34.20	0.97

**TABLE 2.** Model, optimizer, loss, and data settings for experiments.

Model	Optimizer	Loss	Data processing	Method
ResNet18 [26] EfficientN et-B1[27]	Adam [28], SGD [29]	MSE, MAE, MAPE, custom loss	denoising, cropping, normalizing, resizing	Linear probing, Fine tuning

**TABLE 3.** Performance of training settings on FM predictions. BAQ protocol, 3 images each.

Architecture	Learning	Loss	MAE	MSE	RMSE	MAPE
ResNet18	Linear Probing	MAE	0.0701	0.0062	0.0789	48.10%
		MSE	0.0449	0.0028	0.0522	29.18%
	Finetuning	MAE	0.0834	0.0086	0.0924	61.79%
		MSE	0.0712	0.0074	0.0857	48.52%
**EfficientNet-B1**	Linear Probing	MAE	0.1637	0.0289	0.1699	101.63%
		MSE	0.1235	0.0180	0.1341	74.46%
	**Finetuning**	**MAE**	**0.0455**	**0.0032**	**0.0567**	**25.32%**
		MSE	0.1222	0.0174	0.1319	73.28%

**TABLE 4.** P-values from one-sided T-test comparing mean performance metrics across different model configurations (a matrix of p-values indicates pairwise statistical comparisons between different deep learning models and training strategies, based on two evaluation metrics: MAE and MSE. The models evaluated are ResNet18 and EfficientNet, each trained using two approaches: Linear Probing (LP) and Fine-Tuning (FT). Each model-strategy-metric combination is labeled accordingly (e.g., ResNet18_LP_MAE, EfficientNet_FT_MSE).

	B	A	Q	AB	BQ	AQ	BAQ
FM	43.28%	164.08%	187.37%	60.70%	**26.10%**	73.63%	**25.32%**
FFM	80.75%	92.71%	74.31%	86.07%	81.02%	85.57%	82.15%

**TABLE 5.** MAPE Results for combinations of anatomical image locations.

	ResNet18 _LP_MAE	ResNet18 _LP_MAE	ResNet18 _FT_MAE	ResNet18 _FT_MSE	EfficientNet _LP_MAE	EfficientNet _LP_MSE	EfficientNet _FT_MAE	EfficientNet _LP_MSE
ResNet18_LP_MAE	NA	1	2.93E-49	3.79 E-32	9.12 E-64	2.01 E-52	1	2.64 E-56
ResNet18_LP_MSE		NA	3.54E-49	3.46 E-35	9.93 E-63	4.69 E-56	1	5.57 E-56
ResNet18_FT_MAE			NA	1	7.01 E-48	2.90 E-26	1	6.02 E-28
ResNet18_FT_MSE				NA	1.16 E-52	6.74 E-40	1	5.91 E-40
EfficientNet_LP_MAE					NA	1	1	1
EfficientNet_LP_MSE						NA	1	1
EfficientNet_FT_MAE							NA	2.39 E-58
EfficientNet_LP_MSE								NA
